# Proteomics in non-human primates: utilizing RNA-Seq data to improve protein identification by mass spectrometry in vervet monkeys

**DOI:** 10.1186/s12864-017-4279-0

**Published:** 2017-11-13

**Authors:** J. Michael Proffitt, Jeremy Glenn, Anthony J. Cesnik, Avinash Jadhav, Michael R. Shortreed, Lloyd M. Smith, Kylie Kavanagh, Laura A. Cox, Michael Olivier

**Affiliations:** 1 Department of Genetics, Texas Biomedical Research Institute, San Antonio, Texas USA; 2 Department of Chemistry, University of Wisconsin, Madison, Wisconsin USA; 3 Genome Center of Wisconsin, University of Wisconsin, Madison, Wisconsin USA; 4 Department of Pathology and Comparative Medicine, Wake Forest School of Medicine, Winston-Salem, North Carolina USA; 5 Southwest National Primate Research Center, Texas Biomedical Research Institute, San Antonio, Texas USA; 6 Current address: Department of Internal Medicine, Section of Molecular Medicine, Wake Forest School of Medicine, NRC Building, G-55, Winston-Salem, North Carolina 27157 USA

**Keywords:** Proteogenomics, Proteomics, Liver, Vervet, RNA-Seq, Morpheus, Non-human primate, Galaxy-P

## Abstract

**Background:**

Shotgun proteomics utilizes a database search strategy to compare detected mass spectra to a library of theoretical spectra derived from reference genome information. As such, the robustness of proteomics results is contingent upon the completeness and accuracy of the gene annotation in the reference genome. For animal models of disease where genomic annotation is incomplete, such as non-human primates, proteogenomic methods can improve the detection of proteins by incorporating transcriptional data from RNA-Seq to improve proteomics search databases used for peptide spectral matching. Customized search databases derived from RNA-Seq data are capable of identifying unannotated genetic and splice variants while simultaneously reducing the number of comparisons to only those transcripts actively expressed in the tissue.

**Results:**

We collected RNA-Seq and proteomic data from 10 vervet monkey liver samples and used the RNA-Seq data to curate sample-specific search databases which were analyzed in the program Morpheus. We compared these results against those from a search database generated from the reference vervet genome. A total of 284 previously unannotated splice junctions were predicted by the RNA-Seq data, 92 of which were confirmed by peptide spectral matches. More than half (53/92) of these unannotated splice variants had orthologs in other non-human primates, suggesting that failure to match these peptides in the reference analyses likely arose from incomplete gene model information. The sample-specific databases also identified 101 unique peptides containing single amino acid substitutions which were missed by the reference database. Because the sample-specific searches were restricted to actively expressed transcripts, the search databases were smaller, more computationally efficient, and identified more peptides at the empirically derived 1 % false discovery rate.

**Conclusion:**

Proteogenomic approaches are ideally suited to facilitate the discovery and annotation of proteins in less widely studies animal models such as non-human primates. We expect that these approaches will help to improve existing genome annotations of non-human primate species such as vervet.

**Electronic supplementary material:**

The online version of this article (doi: 10.1186/s12864-017-4279-0) contains supplementary material, which is available to authorized users.

## Background

Shotgun proteomic approaches employ a database search strategy to compare experimentally observed mass spectra to an in silico-generated library of theoretical spectra derived from gene annotation information of the organism(s) being studied. The successful matching of peptides is thus predicated upon the accuracy of the search database being utilized to make these comparisons. The outcome of proteomics experiments is therefore driven by the quality and completeness of the genomic information of the organism being studied. Proteomic studies of genetically well-characterized species such as mice and humans benefit from robust proteomic search databases and extensive genome annotations which can account for known genetic variability such as splice variants and sequence variation altering the amino acid sequence of encoded proteins. However, protein identification of other research model organisms is limited by the quality of reference genome annotations.

Proteogenomic methods attempt to improve the search library limitations by leveraging information about gene transcription to guide the curation of search databases customized to the tissues of individual organisms. Several groups have demonstrated that transcriptional profiling using massively parallel sequencing approaches (RNA-Seq) improves the detection of peptides in proteomics experiments in a wide range of different species, ranging from microorganisms [1–3] and plants [4–7] to crustaceans [8], squids [9, 10], honey bees [11], chicken [12], ground squirrels [13], pig [14], and sheep [15]. The approach improves peptide assignment primarily in three important ways [16–18].

First, RNA-Seq data reveal sample-specific genetic sequences which may differ from the reference genome including nucleotide insertions, deletions, or substitutions. Single nucleotide polymorphisms (SNPs) comprise the majority of genomic variation within coding exons of genes, and these genetic variants are divided into two broad categories; SNPs which change a coding triplet but do not result in an amino acid substitution are referred to as synonymous SNPs, while variants which result in amino acid substitutions are called non-synonymous SNPs (nsSNPs). The identification and inclusion of nsSNPs has the potential to improve the search database annotation because these changes can alter the chemical properties of the fragmented peptides. Failure to account for the resultant mass and/or charge change arising from the amino acid substitutions introduces ambiguity in peptide matching because, unlike nucleotide sequencing where the base order and fragment size can be directly inferred from the raw data, proteomic matching of peptides is based solely on the atomic mass and charge expected to be derived from enzymatically fragmented proteins.

Second, RNA-Seq reads can be used to identify splice junctions (SJs) which characterize mRNA isoforms absent from the reference gene model. SJs can result from genetic variation that alters how the spliceosome interacts with mRNAs, or SJs might arise as a result of alternative exon usage in tissue- or condition-specific contexts. RNA-Seq can also detect chimeric RNAs which arise from gene fusion events. Search databases that inadequately account for this isoform variability will fail to accurately identify their translated peptide products, and organisms with incomplete gene model information are therefore more susceptible to peptide misidentification.

Finally, RNA-Seq reads can be used to estimate transcript abundance. Knowledge of which mRNAs are expressed can inform how search databases can be trimmed to minimize multiple testing that occurs within peptide spectral matching algorithms. As with any iterative comparison process, the likelihood of misidentifying peptides increases with the total number of comparisons made. Experiments which incorporate proteomics results are vulnerable to Type I error inflation because multiple comparisons are made first at the peptide spectral matching identification stage, and then again in the context of the experimental condition (e.g. identifying protein abundance differences between case and control groups).

Based on this summary, it is evident that sample-specific search databases derived from RNA-Seq analyses from the same tissue sample as the proteomics data provide an improved search database, as it will include those peptides mostly likely to be found within the tissue being sampled, while excluding records derived from extraneous genomic information. This approach becomes even more valuable for analyses in samples from species with poorly annotated genomes [19], for reasons we discuss below.

Several bioinformatics pipelines have been described which facilitate the conversion of RNA-Seq reads into customized peptide databases which can be used to search mass spectrometry (MS) data. A well-established approach, developed and described by Sheynkman et al. [16], leverages the web interface of the Galaxy bioinformatics project [20] to facilitate the coordination of independent bioinformatics tools into a functional, user-generated proteogenomic workflow. As part of our analysis, we specifically implemented the proteogenomics approach using the analysis program Morpheus [21] which is computationally less demanding than other programs, and effectively calculates empirical false discovery rates (FDR) for peptide matches. We utilized this approach to determine whether RNA-Seq data from vervet monkey liver samples, a non-human primate without a well-characterized and annotated genome, improves the detection of peptides in vervet liver proteomic data.

While several non-human primate animal models for disease have been extensively used for decades, only recently have the genomes of these organisms begun to be characterized. The African green monkey, or vervet monkey (*Chlorocebus aethiops sabeus*), is one such example. The vervet monkey has long been an important model in AIDS research, as vervets are natural carriers of SIV yet display no symptoms of illness upon infection [22, 23]. More recently, vervets have provided insight into neurologic [24, 25] and metabolic diseases [26–29]. With the recent release of the first vervet genome [30], this animal model is ideally suited to benefit from the expansion of its genome annotation that proteogenomic approaches can provide. In this paper, we utilize matched RNA-Seq and MS data from vervet liver samples to characterize the vervet liver proteome and demonstrate that proteogenomic methods improve the detection of peptides otherwise missed by search databases constructed from the current reference vervet genome annotation.

## Methods

### Sample collection

All experimental procedures involving animals were approved and complied with the guidelines of the Institutional Animal Care and Use Committee of Wake Forest University Health Sciences and conducted in AAALAC approved facilities. All animals included in this study were female vervet/African green monkeys (*Chlorocebus aethiops sabaeus*) from the Vervet Research Colony (VRC) at Wake Forest School of Medicine. All monkeys were US-colony born within the VRC, which is a multi-generational, pedigreed, and genotyped colony originally founded in 1975 by the University of California Los Angeles, with 57 animals imported from St. Kitts and Nevis. In early 2008, the VRC was transferred to Wake Forest School of Medicine and remains a continuously NIH-supported national research resource. To obtain the samples reported here, 10 vervet monkeys were sedated with ketamine (15 mg/kg intramuscularly), intubated, and anesthetized using isoflurane to facilitate the surgical retrieval of liver tissue via laparotomy. Liver tissue was immediately frozen in liquid nitrogen and stored at –80C until analysis.

### RNA-Seq

Total RNA was extracted from vervet monkey livers using the Zymo Direct-zol™ kit (Zymo Research, R2070) and each sample was subsequently quantified by Qubit assay (Thermo Fisher, Q32852). RNA-Seq libraries were prepared from 500 ng of total RNA according to the Illumina TruSeq stranded mRNA protocol (Illumina, RS-122-2101), which specifically retains polyadenylated mRNAs through the use of oligo dT coated magnetic beads. Sequencing library concentrations were quantified using the KAPA library quantification kit (Kapa Biosystems, KK4824). Clusters were generated by cBot (Illumina), and 2 × 100 base paired-end sequencing libraries were sequenced using the Illumina HiSeq 2500 with v3 sequencing reagents (Illumina, FC-401-3001).

### Conversion of RNA-Seq data to customized peptide databases in galaxy-P

Methods for converting the RNA-Seq reads into searchable protein databases have been extensively described previously [16, 31, 32]. We adapted these approaches within Galaxy-P to create sample-specific search databases for each of the 10 vervet monkey liver samples, using the reference vervet monkey genome (ChlSab1.1) as the basis for the sequence alignments. General overviews of each component of the database construction, along with URLs pointing to the specific workflows with the Galaxy toolshed, are outlined below. Upon completion of the three workflows for each RNA-Seq sample, the records from the three pipelines were concatenated to create a completed sample-specific search database for each of the 10 animals in the study.

### Single amino acid variant (SAV) database construction and workflow

Within the SAV workflow, RNA-Seq reads from one sample are aligned to the vervet reference genome using Tophat [33], single nucleotide variant calls are made using SAMtools [34], and the subset of identified SNPs which reside within exons are subsequently annotated using SnpEff [35]. A tool developed within Galaxy-P called “SNPeff to Peptide Fasta” is used to convert the nucleotide sequences into the expected corresponding amino acid sequences. The complete workflow can be found here: http://toolshed.g2.bx.psu.edu/view/galaxyp/proteomics_rnaseq_sap_db_workflow.

### Splice junction (SJ) database construction and workflow

The SJ workflow begins by aligning the RNA-Seq reads to the reference vervet genome as well as the Ensembl gene models for the species. The coordinates of all the detected junctions are compared between the two, and only those junctions mapping to the reference genome but not the Ensembl gene model are retained for the SJ annotation. The Galaxy-P program “Translate BED sequences” is used to convert the SJs identified by the RNA-Seq reads into the corresponding polypeptide sequences. Full details are available here: http://toolshed.g2.bx.psu.edu/view/galaxyp/proteomics_rnaseq_splice_db_workflow.

### Transcript abundance-based database reduction workflow

In order to reduce the records of proteins based on transcript abundance, RNA-Seq data is quantified by RSEM [36] within the Galaxy-P framework. Quantitative values are normalized and output in transcripts per million (TPM). Text manipulation tools in Galaxy concatenate the protein FASTA data with the transcript identifiers and TPM values, and all records where the values are less than one TPM are excluded from the search database, in accordance with our standard RNA-Seq quality control procedures. Including transcripts with lower abundance increases false-positive alignments, and would require validation through deep sequencing to confirm the presence of the transcript. The workflow repository with the Galaxy toolshed is listed here: http://toolshed.g2.bx.psu.edu/view/galaxyp/proteomics_rnaseq_reduced_db_workflow.

### MS-based proteomics

Proteins were extracted from liver tissue using RIPA lysis buffer, and separated on 4–12% gradient Bis-Tris gel. Three gel slices were excised and each was reduced with 10 mM DTT for 30 min at room temperature and alkylated with 55 mM iodoacetamide in 100 mM ammonium bicarbonate for 30 min at room temperature. The gel pieces were subsequently washed with ultrapure 100 mM ammonium bicarbonate, dehydrated with 100% acetonitrile, and dried by Speedvac for 2–3 min.

Samples were then digested with trypsin (Promega, V5280) at 37 °C overnight. Formic acid (1%) was added to the trypsinized samples to quench the proteolysis, and the peptides were desalted and concentrated using C_18_ ZipTips (Millipore, Z720046-960EA). HPLC separation was performed on a 15 cm column of 3 μm diameter which was packed in house with C_18_ beads. Peptides were loaded onto the column at a flow rate of 400 nl/min for 3 h and MS data were acquired by a data dependent scanning on the Thermo Scientific Orbitrap Elite mass spectrometer utilizing a default top 15 method.

Raw mass spectrometry (MS) files were subsequently analyzed in the program Morpheus [21]. The following settings were used in all searches: Assumed Precursor Charge States, Minimum = 2; Assumed Precursor Charge States, Maximum = 4; MS/MS Peak Filtering, Maximum Number of Peaks = 400; MS/MS Analysis, Assign Charge States = enabled; Protease = trypsin (no proline rule); Maximum Missed Cleavages = 2; Initiator Methionine Behavior = variable; Fixed Modifications = carbamidomethylation of C; Variable Modifications = oxidation of M; Maximum Variable Modification Isoforms Per Peptide = 1024; Precursor Mass Tolerance = ± 2.1 Da (monoisotopic); Precursor Monoisotopic Peak Correction = disabled; Product Mass Tolerance = ± 0.025 Da (monoisotopic); Maximum False Discovery Rate = 1%.

For each liver sample, two sets of Morpheus output files were created; the first analysis was searched using the reference vervet monkey database and the second analysis was searched utilizing the sample-specific database created by the Galaxy-P pipelines described above.

### Comparative proteomic analyses

Prior to comparison of the proteomic results, the six sets of output files from the Morpheus program for each of the liver samples (3 fractions per sample, run against 2 search databases = 6 files/sample) were combined and transformed to create unique identifiers for all of the peptide spectral match records. This permits the direct comparison of spectra matched from the raw MS files. The search database file size comparisons and wall clock times were extracted from the Morpheus summary files. The “VennDiagram” package in R (https://CRAN.R-project.org/package=VennDiagram) was used to create the lists of unique peptides and protein groups, as well as Venn diagram image files. An R markdown document outlining the tidying and concatenation of the Morpheus output files, along with the creation of the Venn diagrams, can be found in Additional file 1. Gene set enrichment analyses were conducted to identify classes of proteins overrepresented within the list of proteins identified by the reference database but not the sample-specific databases [37, 38].

## Results

### Search databases curated from RNA-Seq data are smaller and computationally more efficient than reference genome databases

To demonstrate the utility of RNA-Seq derived proteomics search databases, we created sample-specific databases (SSdb) for each liver sample from 10 different vervet monkeys based on sequenced mRNA extracted from the same tissue sample as the protein being analyzed by MS. As outlined above, this procedure creates a unique optimized search database for each sample from the RNA-Seq data, and each MS dataset for a given sample is searched against just the SSdb. For each of the 10 samples, the peptide spectral matching performance was compared between the SSdb and a search database created from the reference vervet genome (REFdb). The descriptive statistics for the RNA-Seq alignments and MS/MS raw data are outlined in Table 1. The RNA-Seq read depth ranged from 6.5 million to 10.5 million mapped reads for the 10 samples. Despite this variability, we found no relationship between RNA-Seq read depth and peptide spectral matches (PSMs) or unique peptides identified in the samples when searched by the SSdb.

**Table 1:** Descriptive statistics for the RNA-Seq and mass spectrometry analyses utilizing the Vervet reference search database (REFdb, 19,255 gene entries) and the sample-specific databases (SSdb)

Sample	RNA-Seq		SSdb Entries		Mass Spectra	PSMs		Peptide IDs	
	RNA-Seq reads	% reads aligned	Genes	Novel SJs		REFdb	SSdb	REFdb	SSdb
1030	7,040,525	55.5	13,804	4069	80,003	26,525	26,680	9765	9702
1211	6,585,341	68.8	15,782	7171	79,381	27,288	27,673	10,532	10,527
1238	6,594,936	67.1	15,659	6595	78,444	19,600	19,898	9349	9354
1245	6,730,432	64.0	13,901	4089	80,281	29,143	29,193	10,503	10,463
1248	10,504,974	69.4	15,513	7429	80,221	22,205	22,479	9120	9162
1254	9,127,588	62.5	15,936	7641	79,675	23,655	23,738	9334	9385
1291	6,575,182	67.9	13,354	3653	79,960	30,623	30,722	11,478	11,652
1347	6,637,842	56.6	13,284	3147	78,791	17,284	17,037	8633	8575
1448	8,019,158	65.0	15,668	6419	71,853	15,612	15,561	7582	7593
1467	9,983,615	66.2	16,176	7305	78,781	20,101	20,162	9177	9223

Restricting the size of SSdb to transcripts with an abundance of 1 TPM or more condensed the search database size and Morpheus compute time when compared to the REFdb. On average, the SSdbs were 77% of the size of the REFdb, and compute times in Morpheus averaged 53% faster for SSdbs compared to the REFdb (defined by ([REFdb time/SSdb time]-1)).

Interestingly, for two samples (samples 1347 and 1448), the search against the REFdb resulted in more peptide spectral matches (aon average 0.6%) when compared to the search using the SSdb. Similarly, for three samples (samples 1030, 1245, and 1347) the analysis against the REFdb identified slightly more peptides compared to the search using the SSdb (on average 0.5%). Given the lower number of RNA-Seq reads or mass spectra obtained for some of these samples, it is conceivable that this difference is due to variation in sample preparation or sample quality. Partial degradation of tissue samples would affect both RNA and protein recovery, and may have impacted the analyses presented here.

### RNA-Seq derived search databases identify peptides not annotated in the vervet reference genome

**Fig. 1:**
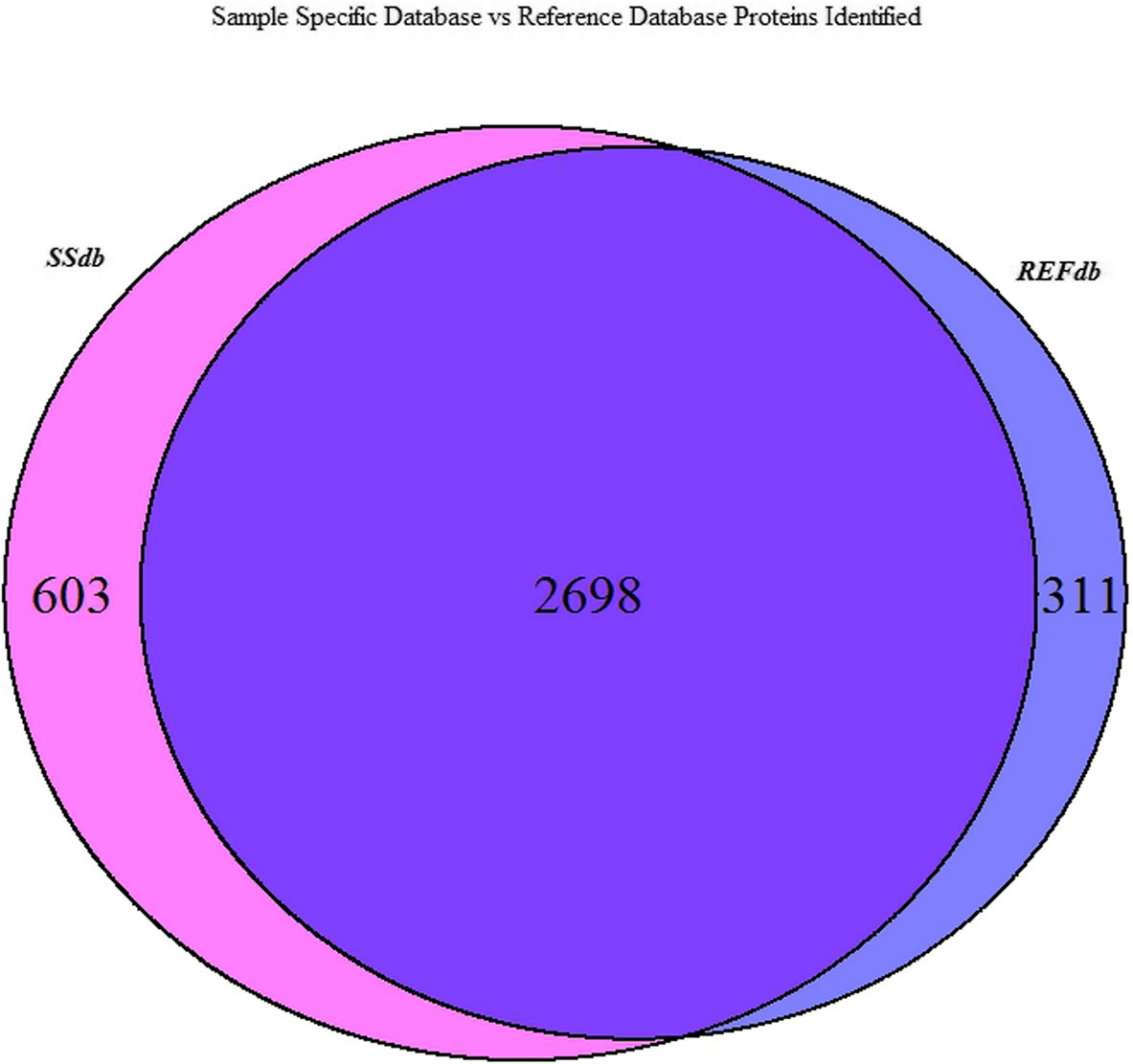
Sample specific database vs reference database proteins identified

Next, we combined the search results across the 10 samples to compare the unique peptides and protein groups identified in vervet monkey liver samples by the REFdb versus the SSdb. These results are shown in the Venn diagram of Fig. 1. We identified 601 peptides in analyses using the SSdb that were not identified using the REFdb.

The first set of these peptides represents peptides that match newly identified SJs not annotated in the gene models of the reference genome. Of the 284 SJ peptide search records identified and annotated from the RNA-Seq data, we identified 47 peptides by MS in more than one sample, which suggests these matches represent incompletely annotated genes of the vervet genome. Consequently, the results of these proteomics analyses could aid in the improved annotation of the gene models. Another 45 peptides mapped to a single sample, bringing the total number of distinctly SJ-mapped peptides identified in these samples to 92. A comparative analysis of these peptide sequences with other primates utilizing BLASTP revealed that the majority of the identified peptides (53/92) could be matched with an orthologous protein [39]. The complete catalogue of SJs with their corresponding peptides can be found in Additional file 2.

A second set of peptides uniquely identified in searches using the SSdb are peptides that map to an SAV record where the amino acid variant resides within the predicted tryptic peptide that matched in the search. In total, 192 peptide matches representing 101 distinct peptides were found in the 10 samples by the sample-specific analyses using the SSdb. Of these 101 distinct peptides that conformed SAVs identified in the RNA-Seq data, 37 peptides were identified in more than one sample. A list of all peptides matching SAV records are included in Additional file 2.

These first two categories of peptides identified within the SSdb analyses represent search results we expected to obtain from the RNA-Seq-based proteogenomic approach because the mRNA read data create search records which accurately predict the respective peptide fragments seen in the MS dataset.

### Reduction of the search database size recovers peptide identifications

While we might expect the same number of matches to reference protein entries contained in both the SSdb and REFdb searches, a total of 313 peptides were identified as matches to reference proteins in the search against the SSdb but not the REFdb. Morpheus, like many other spectral matching algorithms, uses a decoy-based searching approach to empirically estimate and maintain a 1% false discovery rate (FDR). The inclusion of these peptides may result from the adjustment of the absolute FDR that arises from the reduction in the absolute size of the SSdb compared to the REFdb. This is supported by the fact that the q-values of this subset of SSdb-identified peptides is higher, on average, compared to the overall average q-value of all peptides identified using the SSdb search. Of the “reference” peptides identified using the SSdb, only 16% (151 of 948) were seen in two or more samples. The peptides identified exclusively in the SSdb analyses are listed in Additional file 3.

### Peptide identifications missed by RNA-Seq-derived databases are predominantly structural proteins

While the minimization of the search database helps recover some true positive matches, as described above, concerns about restricting proteome search databases based upon RNA-Seq data could arise when a protein’s abundance is poorly correlated with its corresponding transcript abundance, or when proteins might be derived from a tissue of origin different from which they currently reside. Extracellular structural proteins or chromatin-associated proteins with long-half-lives would be likely candidates for the former category, while growth factors, cytokines or contaminating proteins might comprise the latter. We surveyed the list of peptides identified by REFdb but not SSdb analyses and identified 1950 total matches mapping to 891 unique peptides, of which 506 (57%) were identified in 2 or more liver samples. Those 506 peptides correspond to 238 unique protein entries in the vervet ENSEMBL database. Gene set enrichment analysis revealed that this list of proteins was indeed overrepresented by structural, cytoskeletal, and ribonucleotide binding proteins. Table 2 lists the significantly enriched categories identified in this subset of proteins. The peptides identified by the REFdb but not the SSdbs searches can be found in Additional file 4.

**Table 2:** Gene Set Enrichment Analysis for proteins identified by reference but not sample-specific databases

GO Annotation	Description	*p*-value	FDR q-value
GO: STRUCTURAL MOLECULE ACTIVITY	The action of a molecule that contributes to the structural integrity of a complex or assembly within or outside a cell.	3.03 × 10^−15^	3.42 × 10^−11^
GO: OXIDATION REDUCTION PROCESS	A metabolic process that results in the removal or addition of one or more electrons to or from a substance, with or without the concomitant removal or addition of a proton or protons.	9.8 × 10^−14^	5.52 × 10^−10^
GO: DNA PACKAGING COMPLEX	A protein complex that plays a role in the process of DNA packaging.	7.61 × 10^−13^	2.86 × 10^−9^
GO: EXTRACELLULAR SPACE	That part of a multicellular organism outside the cells proper, usually taken to be outside the plasma membranes, and occupied by fluid.	1.25 × 10^−11^	2.81 × 10^−8^
GO: PROTEIN DNA COMPLEX	A macromolecular complex containing both protein and DNA molecules.	6.04 × 10^−11^	1.13 × 10^−7^

Though distinct differences in the peptides identified by the SSdb versus REFdb have been revealed by this comparative exercise, it is perhaps equally important to highlight that SSdb analyses are capable of matching nearly 98% of the same spectra as the REFdb. The customized databases created from RNA-Seq robustly identify proteins from tissue samples, identify peptide variation that would otherwise be missed by searching against the reference annotation, and perform these functions using smaller file sizes and faster compute times.

## Discussion

This work represents the implementation and proof-of-concept application of an established RNA-Seq proteogenomic approach to improve the identification of proteins in non-human primate proteomics experiments, such as the vervet monkey liver sample analyses reported here. Several non-human primate genomes have been drafted but incompletely annotated because a limited number of animals have been sequenced. Less common sequence and splice variants will continue to be incorporated as DNA sequencing sample sizes increase and more complete transcriptional profiles across tissue types are reported. In the meantime, a proteogenomic approach should improve protein identification in MS experiments in these species, and may offer robust and reliable data to improve the existing annotation using both transcript and protein data. We demonstrated that this analysis approach, implemented in Galaxy-P, is capable of identifying peptides derived from unannotated splice junctions and non-synonymous coding substitutions revealed from the RNA-Seq read data. These peptides would have gone unmatched by searching the MS data using the vervet reference genome annotation data. These novel peptides likely represent a combination of common, yet previously unannotated gene isoforms as well as isoforms and variants private to individual animals studied.

As with other “omics” scale analyses, the iterative search process of shotgun proteomics presents challenges in balancing type I and type II error rates. The optimal search database would incorporate only those potential proteins which are likely to be found within a given sample; however, if the repertoire of proteins were already known, it would obviate the need for conducting proteomics experiments in the first place. Instead, RNA-Seq data can facilitate a compromise in the database curation process by predicting non-reference protein isoforms for inclusion while also utilizing information about transcript abundance to exclude individual gene sequences from the search records as the corresponding mRNA are not expressed and therefore the translated protein (or a peptide thereof) is unlikely to be identified by MS. While RNA-Seq data can be a useful benchmark for restricting the size of the MS search database, it should be noted that certain exceptions to the database exclusion process should be considered. Examples include proteins whose tissue of origin is different from the sampled tissue, such as blood-derived albumin, immunoglobulins, and complement proteins, or long-lived structural proteins such as collagen or ribosomal proteins, whose protein abundance is uncoupled from their corresponding mRNA expression in the tissue of interest at the time of sample collection. By comparing peptide matches made using RNA-Seq derived databases to peptides identified using the reference database, we have revealed a list of proteins routinely found in vervet livers that do not have corresponding mRNA abundance levels from RNA-Seq read data. Including these protein records in the construction of sample-specific databases for liver samples could mitigate the loss of information in future proteomics experiments.

The reduction of the search database size and the included protein records significantly impacts the confidence with which peptides and proteins are identified. Due to the smaller number of records to compare an experimental spectrum to, the confidence with which individual peptides are assigned to the correct record is higher. However, these “improved” matches have another consequence: some experimental peptide spectra may not be confidently assigned to a specific sequence in the REFdb analysis since the match may not fall under the stringent 1% FDR commonly required to confirm a match. However, using the smaller SSdb, the same match is now made with a FDR of less than 1% (simply based on the smaller number of searches), resulting in some additional “reference” proteins being identified in the SSdb searches at a 1% FDR but not in the REFdb searches. It is possible that these matches may include some low confidence peptide matches, but overall these additional peptide matches emphasize the additional power that is gained from reducing the search database to only relevant records of expressed proteins and peptide sequences.

Our analysis only used one standard established analysis approach for these comparisons. The analysis pipeline implemented in Galaxy includes the Morpheus search algorithm which empirically calculates the FDR for peptide matches obtained in the search. Numerous other approaches have been proposed for the analysis of proteogenomic data, and the assessment of FDR in peptide and protein identifications. It is likely that some of these approaches, such as analyzing FDR separately for REFdb matches and matches to novel SSdb records derived from RNA-Seq [19] or alternative programs to calculate FDR in these datasets [40–42], would improve the results presented here, and further enhance the utility of this proteogenomic approach for non-human primate proteomics. However, a detailed comparison of these different analysis approaches was not the goal of the current study, and future studies will help define the optimal approach for a proteogenomic analysis in these species, including the optimal RNA-Seq coverage and the depth of proteomic analysis. Prior analyses have generated far more detailed mass spectral analysis data (500,000 mass spectra compared to 80,000 used in our study), and it remains to be seen what the optimal approach will be [16]. As proteogenomic approaches continue to gain momentum in shotgun proteomics experiments, we anticipate further refinement of search databases to account for biochemical variability in peptides which arise from post-translational modifications (PTMs). A recent publication has outlined an approach to parsimoniously account for peptide mass shifts caused by PTMs through incorporating Uniprot annotation data ([43, 44]). Similarly, proteogenomics can incorporate findings from complimentary NGS approaches, such as ribosomal profiling, to expand the prediction of the protein-coding products from novel coding sequences [45] and lncRNA molecules previously presumed to be untranslated [44, 46]. Continued refinements to search databases and proteomics search algorithms will accelerate the accurate identification and quantification of peptides in MS analyses, and it will complement and improve the genome annotation of animal research model organisms and help researchers utilize shotgun proteomics to implicate protein changes associated with pathophysiologic processes. Ultimately, the proteomic validation of novel splice variants and non-synonymous sequence variants will greatly enhance the ongoing efforts of genome annotation, especially in model species with poorly annotated genomes, such as many non-human primates.

## Conclusions

A proteogenomic approach to the analysis of liver shotgun proteomic data from a nonhuman primate species, the vervet/African green monkey (*Chlorocebus aethiops sabaeus*), demonstrates that the use of sample-derived RNA-Seq data, as anticipated, improves peptide identification and the accuracy and confidence of protein identification, while simultaneously reducing the search database space and the resulting computing effort required for the data analysis. Novel peptides including sequence variants identified by RNA-Seq, as well as new splice variants uncovered in the transcriptional analysis account for the majority of the novel peptides identified, highlighting the importance of proteogenomic approaches in species with limited available genome sequence data and gene annotation, such as non-human primates.

## Additional files

Additional file 1: Outlining the data transformations and analyses of the Morpheus file outputs. (HTML 641 kb)

Additional file 2: Listing the SAV and SJ derived peptides identified exclusively by the sample-specific databases. (XLSX 46 kb)

Additional file 3: Listing the reference genome peptides identified exclusively by the sample-specific databases. (XLS 343 kb)

Additional file 4: Listing the peptides identified by the REFdb but not the SSdb searches. (XLS 541 kb)

## Abbreviations

AIDS: Acquired immune deficiency syndrome
DTT: Dithiothreitol
FDR: False discovery rate
GO: Gene ontology
GSEA: Gene set enrichment analysis
HPLC: High performance liquid chromatography
lncRNA: Long, non-coding ribonucleic acid
mRNA: Messenger ribonucleic acid
MS: Mass spectrometry
PSM: Peptide spectral match
REFdb: Reference database
SAV: Single amino variant
SIV: Simian immunodeficiency virus
SJ: Splice junction
SNP: Single nucleotide polymorphism
SSDb: Sample-specific database
TPM: Transcripts per million
