# Supplementary material for: Proteomics in non-human primates: utilizing RNA-Seq data to improve protein identification by mass spectrometry in vervet monkeys
Source: BMC Genomics. 2017 Nov 13;18:877. doi: 10.1186/s12864-017-4279-0 (PMC5683380; doi:10.1186/s12864-017-4279-0)
Supplement: Supplementary file 1 — Outlining the data transformations and analyses of the Morpheus file outputs. (HTML 641 kb) [file 12864_2017_4279_MOESM1_ESM.html]

Vervet proteomic Supplement S1


# Vervet proteomic Supplement S1

#### *Mike Proffitt*

#### *September 28th, 2016*

This is an R Markdown document. Markdown is a simple formatting syntax for authoring HTML, PDF, and MS Word documents. For more details on using R Markdown see http://rmarkdown.rstudio.com.

When you click the **Knit** button a document will be generated that includes both content as well as the output of any embedded R code chunks within the document. You can embed an R code chunk like this:

```
summary(cars)
```

## Introduction

I’ve created this R markdown document to keep track of the work I’m doing with the 10 vervet proteomic samples we have run in Michael Olivier’s lab as part of a collaboration with Laura Cox at TBRI and Kylie Cavanaugh from Wake Forest. The 10 animals constitute a case control study where 5 animals were exposed to a long-term high sugar feeding challenge and 5 control animals remained on standard chow. Blood, urine, and liver samples were obtained following the challenge. The liver samples were sent to Texas Biomed, where Laura Cox’s lab performed RNAseq on the samples. Michael’s lab conducted proteomic profiling on these same liver samples. Jerry Glen from Dr. Cox’s lab, working with Anthony Cesnik from UW-Madison, implemented a Galaxy-based workflow to create sample-specific proteomic search databases based on the information derived from the RNA-seq reads. I’m taking the resulting FASTA files from that pipeline and feeding them into several proteomic search engines (the Morpheus program among them) for proteomic analysis. Here’s lies my documentation of the “Morpheus-specific”" part of those analyses

The rationale behind using RNAseq data to search mass spec spectra can be broken out into 3 main factors. First, sample-specific databases (SSDs) should improve the false discovery rate by reducing the number of comparisons made between spectra and potential peptides because the RNAseq data from liver should only represent a subset of the total transcriptome. Second, the RNAseq data has the potential to identify unannotated splice variants that may be specific to particular samples. Third, the SSDs should improve the detection of peptides where nonsynonymous coding variants alter the amino acid sequence in such a way that the mass and/or charge of the peptide would not match the reference annotation.

To evaluate the effectiveness and utility of using SSDs to call proteomic mass spectra, we have run the 10 proteomic samples against both a reference peptide library and a SSD for each sample. This R markdown document will be used to follow the manipulation and interrogation of the data derived from the Morpheus runs.

The original files being used are located here: Server18/OlivierLab/Mike. Within the Mike directory, there are 2 folders, named *Vervet Reference runs* and *Vervet SSD runs*. Within each of those folders are folders corresponding to the individual sample IDs. Within those folders, a file named *unique peptides sampleID* along with *ref* or *ssd* will appear. These are the raw files being used in this exercise.

The first exercise will explore which spectra (and corresponding peptide matches) are differentially identified.To accomplish this, we need a way to make “apples to apples” comparisons between the 2 analyses; that is, we need to insure that the same spectrum from the same run is being compared between the two databases.

In order to accomplish this, we need to manipulate the raw files to link the spectra to a particular run. Right now, the data are aggregated across 3 fractions, and there is no way to discern the particular calls without appending information from 2 different variables together. For the sake of convenience, I’ve done this by apending the last 6 characters from the **Filename** variable to the **Spectrum Number** variable. This will create a new variable column called **SpecID** which will concatenate the spectrum with the raw run from which it came. This **SpecID** variable will be used to anchor the observations between the reference and the SSD runs.

We also need to clean up a little more. We are only interested in the spectra that have passed the QC parameters we have set for these analyses. Specifically, the spectra to be considered must be **TRUE** for the **Target.** variable, **FALSE** for the **Decoy.** variable, and the **Q-value** must be **less than 1**.

That R code will look like this for each sample ID:

```
# Getting started. Set working directory. For me, that is C:/Users/mproffit/Desktop/MASTER/Vervet_data because the data is stored locally on my hard drive. 
# Note the "/" convention  the R programming language uses (instead of Microsoft "\") to separate directories

setwd("C:/Users/mproffit/Desktop/MASTER/Vervet_data")

# Load the libraries we will need. 
# Please note, if you don't already have these packages, install them first using install.packages()

library(dplyr)
library(magrittr)
library(stringr)


# Create modified table where the data is sorted and subsetted based on the criteria we talked about above.
# Specifically, we:
#1)read the table, 
#2)filter on target equals true, 
#3)filter on decoy equals false, 
#4)filter on q values less than 1, and then 
#5)rearrange the table by q values in ascending order. 
#6)grab the last 6 characters from the "Filename"" variable  and assign it to a new variable named "Temp", 
#7)create a new variable named "SpecID" by combining the "Spectrum.Number" and the "Temp" variables, 
#8)remove the variables (i.e. columns) "Temp", "Spectrum.ID", and "Spectrum.Title" 
#9)write the modified table to csv for future use
#Each of these steps is separated by the "pipe" function from magrittr, which takes the form %>%. 
#The eight steps are also broken out by row.

# We do this for the reference database searches:

id1030ref <- 
  read.table("C:/Users/mproffit/Desktop/MASTER/Vervet_data/unique_peptides_1030_ref.tsv", sep='\t', header = TRUE) %>% 
  filter(., Target. == "True") %>% 
  filter(., Decoy. == "False") %>% 
  filter(., Q.Value.... < 1) %>% 
  arrange(., Q.Value....)  %>% 
  transform(Temp = str_sub(.$Filename, start=-6)) %>% 
  mutate(., SpecID = paste(Spectrum.Number, Temp)) %>% 
  select(., -Temp, -Spectrum.ID, -Spectrum.Title) 
write.csv(id1030ref, file ="C:/Users/mproffit/Desktop/MASTER/Vervet_data/Cleanpep/refs/clean1030ref.csv")
  

# Then we repeat the same for sample specific database searches:

id1030ssd <- 
  read.table("C:/Users/mproffit/Desktop/MASTER/Vervet_data/1030mod_unique_peptides.tsv", sep='\t', header = TRUE) %>% 
  filter(., Target. == "True") %>% 
  filter(., Decoy. == "False") %>% 
  filter(., Q.Value.... < 1) %>% 
  arrange(., Q.Value....)  %>% 
  transform(Temp = str_sub(.$Filename, start=-6)) %>% 
  mutate(., SpecID = paste(Spectrum.Number, Temp)) %>% 
  select(., -Temp, -Spectrum.ID, -Spectrum.Title) 
write.csv(id1030ssd, file ="C:/Users/mproffit/Desktop/MASTER/Vervet_data/Cleanpep/mod/clean1030ssd.csv")
```

Finally, we are ready to make the comparison between the files. There are several ways to do this, but I have chosen the *anti\_join* function in dplyr

```
# Compare between tables using anti-join based on the SpecID variable;
# write the table of differences based on SpecID

anti_join(id1030ref,id1030ssd, by = "SpecID") %>%
write.csv(., file ="C:/Users/mproffit/Desktop/MASTER/Vervet_data/dif/1030dif2.csv")

# Compare between tables using anti-join based on the "Base Peptide Sequence" variable; 
# Write the table of differences based on Base Peptide Sequence
# remove the temporary files named id1030ref and id1030ssd

anti_join(id1030ref,id1030ssd, by = "Base.Peptide.Sequence") %>%
write.csv(., file ="C:/Users/mproffit/Desktop/MASTER/Vervet_data/difpep/1030difpep2.csv")
rm(id1030ref, id1030ssd)
```

The corresponding output file, with the naming convention **sampleid**dif or **sampleid**difpep will contain the spectra information for those spectra or base peptide sequences identified by the SSD but not the reference. These can be examined in further detail to decipher how they were assigned by the SSD.The outputs have been conducted on a sample-by-sample basis initially to explore the inter-individual variability in SSD outputs and idnetify potential outliers, either from an RNA-Seq or proteomics perspective.

After aggregting and trimming, I have created a single file which contains the unique peptide matches for all the samples, across both the reference and sample-specific database search results. This is the file used to create the Venn diagrams outlined below.

### Here is how we generated the venn diagrams

```
# I have eval= FALSE; that's why you are not seeing the actual diagrams...


setwd("C:/Users/mproffit/Desktop/MASTER/Vervet_data/Cleanpep")

library("gdata")
#get the data we need
proteinlists <- read.csv(file ="C:/Users/mproffit/Desktop/MASTER/Vervet_data/Cleanpep/proteinlist_venn_sep28.csv", sep=",", stringsAsFactors=FALSE, header=TRUE)

#remove blanks and make lists needed for venndiagrams to work
proteinLS <- lapply(as.list(proteinlists), function(x) x[x != ""])

names(proteinLS) <- c("SSD", "REFERENCE")

require("VennDiagram")

Venn.list <- proteinLS

#assign the parameters needed to plot venn diagram
venn.plot <- venn.diagram(Venn.list, 
                          NULL, 
                          fill=c("magenta", "blue"), 
                          alpha=c(0.5,0.5), 
                          cex = 2, 
                          cat.fontface=4,
                          category.names=c("SSdb", "REFdb"), 
                          main="Sample Specific Database vs Reference Database Proteins Identified")

#actually plot the venn diagram

grid.draw(venn.plot)

# create the lists of data needed for export

require("gplots")

a <- venn(Venn.list, show.plot = FALSE
          
venninters <- attr(a,"intersections")

lapply(venninters,head)

#convert the list to flat dataframe structure

asdf <- do.call(rbind, venninters)

#export the lists

write.csv(asdf, file ="C:/Users/mproffit/Desktop/MASTER/Vervet_data/Cleanpep/protein_venn_output_sep28.csv")
```

end
